# Parent-offspring association of metabolic syndrome in the Framingham Heart Study

**DOI:** 10.1186/1758-5996-6-140

**Published:** 2014-12-15

**Authors:** Rumana J Khan, Samson Y Gebreab, Pia Riestra, Ruihua Xu, Sharon K Davis

**Affiliations:** Cardiovascular Section, Genomics of Metabolic, Cardiovascular and Inflammatory Disease Branch, Social Epidemiology Research Unit, National Human Genome Research Institute, 10 Center Drive, Room 7 N316 MSC 1644, Bethesda, MD 20892 USA

**Keywords:** Metabolic syndrome, Parent-offspring, Abdominal obesity, Impaired fasting glucose, Framingham Heart Study

## Abstract

**Background:**

Metabolic syndrome (MetS) is a clustering of five metabolic risk factors including abdominal obesity, elevated blood pressure, hypertriglyceridemia, low high-density lipoprotein cholesterol (HDL-C), and impaired fasting glucose. Few studies have fully reported the strength of clustering of these risk factors in a parent-offspring relationship. This analysis describes the associations between parents and their adult offspring in regard to MetS. It also estimates the association between each risk factor in parents and the presence of MetS in their offspring.

**Methods:**

We analyzed data for 1193 offspring (565 sons, and 628 daughters) from the Framingham Offspring Study who attended examinations 5, 6, and 7. Information about their parents was collected from examinations 13, 14 and 15 of the Framingham Original Cohort study. We used pedigree file to combine parental and offspring’s data. Participants were classified as having the MetS according to the Adult Treatment Panel III criteria. Analyses were conducted separately for mothers and fathers. Logistic regression was used to estimate the associations.

**Results:**

After adjusting for age, education, smoking, alcohol consumption and physical activity level of offspring, no significant association was found between father’s and their offspring’s MetS. Mother’s MetS was significantly and positively associated with their daughter’s MetS (adjusted odds ratio or adj OR: 1.63; 95% confidence Interval, CI:1.02-2.61), but not with their sons’ MetS. When analyzed by individual components, maternal impaired glucose (adj OR: 2.03; 95% CI: 1.02- 9.31), abdominal obesity (adj OR: 1.56; 95% CI: 0.98- 2.55) and low HDL-C (adj OR: 2.12; 95% CI: 1.36-3.32) were associated daughter’s MetS. Maternal low HDL-C and raised total cholesterol showed marginal association with son’s MetS. For fathers, only impaired glucose (adj OR: 4.91; 95% CI: 2.07- 11.68) was associated with their daughter’s MetS.

**Conclusions:**

Using the data from Framingham Heart Study, we demonstrate differential association of MetS and its components between parents and offspring. Mother’s MetS was strongly related with daughter’s MetS, but the association was inconsistent with son’s MetS. No association was found between father’s MetS and offspring’s Mets. These results provide evidence that daughters with mother’s MetS are in higher risk than daughters or sons with father’s MetS.

## Background

Metabolic syndrome (MetS) is a clustering of five metabolic risk factors including abdominal obesity, elevated blood pressure, hypertriglyceridemia, low high-density lipoprotein cholesterol (HDL-C), and impaired fasting glucose (IFG). Adult Treatment Panel III (ATPIII) criteria classifies individuals as having the MetS if they have at least three of the above five risk factors [1, 2]. Different studies have shown the association of MetS and different components of MetS at different familial level, including parent offspring, sibling-sibling, and cousin-cousin relationship [3–7]. Some heritability studies have reported reasonable heritability of MetS and according to various studies, heritability estimates for MetS among Caucasian people range from approximately 14 to 27% [8–10]. Linkage analysis, genome-wide association studies, and epigenetic studies have also identified associated genes related to each of the component of MetS [11, 12]. But as MetS is the combined effect of more than one risk factor, multiple genes are probably involved in its development with varying degree of influence from multiple environmental and social factors [13]. Although studies have shown the familial association and considerable amount of heritability of MetS and its components, few studies have directly and fully reported the strength of clustering of these risk factors in a parent-offspring relationship [5–7]. Most studies on this issue have investigated the associations of MetS and its components between adolescent or young children and their parents [5, 6]. The strength and the pattern of the association of MetS between parents and offspring could differ by offspring’s age since at a young age offspring live at their parents’ home and share the same environment. Therefore, it is important to include a wide age range of the offspring to report the association fully. Furthermore, it is also important to take into account the important socio demographic and lifestyle characteristics that contribute to this relationship. The multigenerational design of the Framingham Heart Study (FHS) provides the unique opportunity to accurately investigate this association. A better understanding of the magnitude and the pattern of these associations will improve health professionals’ ability to reliably predict patient’s cardiovascular risk from reported family history. This study describes the associations between parents and their adult offspring in regard to MetS. It also estimates the association between each risk factor in parents and the presence of MetS in their offspring.

## Methods

### Study population

Data for this study were drawn from the offspring and original cohort of FHS. The original FHS cohort included 5209 residents of Framingham, Mass, aged 28 to 62 years who have been followed up with biennial examinations from 1948 to present [14]. The offspring FHS cohort included 5124 (3548 adult children of the original cohort members and 1576 of their spouses) persons aged 5 to 70 years who have been followed up with examinations every 4 to 8 years from 1971 to present [15]. Both original and offspring FHS cohorts are predominantly white [14, 15]. At enrollment and during each follow up participants of both cohorts completed a structured interview with a detailed medical history and risk behavior assessment, a physical examination, and laboratory and other measurements. The FHS examination protocols are reviewed by the institutional review board of the Boston University Medical Center, Boston, Mass, and all participants signed informed consent. FHS has presently completed 31st Exam for the original cohort and the 8th exam for the offspring cohort.

The current analysis included FHS offspring (age 21 years and above) who attended any of the offspring FHS examinations 5, 6, or 7 (over time period, 1991–2001). They were eligible for the present study if they had at least one parent participated in any of the original FHS exam 13, 14 or 15 (time period, 1972–1979). Using the family pedigrees file, the data from parents and the offspring examinations were combined that provided a full range of parent-offspring cohort. After combining the parent and offspring information using the pedigree data, the final analysis included 1193 adult offspring members (565 male offspring or sons, and 628 female offspring or daughters) from the offspring study who had information about either parent in the original cohort. Out of 1193 participants 897 offspring had information about their mothers, 702 had information about father and 406 had data about both parents.

### Data collection

Information about socio-demographic (age, education and income) and lifestyle characteristics (smoking status, alcohol consumption and physical activity) for the offspring participants were obtained from the 5th examination (over time period, 1991–1997) of the offspring cohort. Data on anthropometry, resting blood pressure, blood glucose level, and lipid profiles for the offspring were collected from the 5th, 6th and 7th examination (over time period, 1991–2001). Their parents’ information was obtained from 13th, 14th and 15th examinations (time period, 1972–1979) of original cohort. We combined the examinations for the offspring and their parents to increase the sample size and to account for the missing values. For both offspring and their parents, measurements from the latest examination were used for analysis. If there was any missing value in the latest examination, measurements from the immediate previous examination was used.

### Assessment of MetS

ATP III criteria were used to define MetS for offspring and parents of FHS [1, 2]. This classifies individuals as having MetS if they have at least three of the following five components: (1) abdominal obesity or large waist circumference (>102 cm for men and > 88 cm for women); (2) high triglyceride levels (fasting plasma triglyceride concentration ≥ 1.7 mmol/l or on drug treatment); (3) low HDL-C levels (<1.03 mmol/l for men and < 1.3 mmol/l in women or on drug treatment); (4) elevated blood pressure (≥130 mm Hg systolic or ≥ 85 mm Hg diastolic or on drug treatment); or, (5) impaired fasting glucose or IFG (≥6.1 mmol/l or on drug treatment).

For parents, data for waist circumference was not available. We used body mass index or BMI (weight in kg/height in meter square) as a substitute. BMI more than or equal to 30 was used as a cut off to define obesity. As the blood samples of parents were collected from non-fasting state, IFG was considered present if the random blood glucose level was ≥ 11.1 mmol/l or if they used insulin or oral hypoglycemic agents [16]. For the same reason, triglyceride values was also not used, instead high blood cholesterol or total cholesterol level ≥ 6.20 mmol/l and/ or use of cholesterol-lowering medications was used as a proxy [1].

### Assessment of covariates

Information on the following covariates from the offspring FHS were collected: age in years, sex, education (< high school, high school, college graduate or higher), total family income per year (< $20,000, $20,000- $44,000, $45,000 or higher), physical activity, smoking status and alcohol consumption. Physical activity was assessed with a questionnaire, and was based on the average daily number of hours of sleep and sedentary, slight, moderate, and heavy activity of the participant. The composite score was calculated by summing the number of hours spent in each activity intensity level and multiplying by a respective weight factor derived from the estimated oxygen consumption [17]. Participants were considered smokers if they had smoked regularly in the previous year, otherwise they were categorized as non-smokers. Alcohol use was dichotomized on the basis of consumption of at least one drink per week.

### Analysis

Gender specific characteristics (socio-demographic status, lifestyle characteristics, physical and laboratory measurements) of participants were obtained and were compared using the chi-square test or independent t test. The primary outcome measure for this analysis was presence of MetS among offspring evaluated as a dichotomous variable. And the main predictor was parental MetS (either in both parents or in any parent). Analyses were conducted separately for mothers and fathers. Logistic regression was used to examine the association between offspring and parental MetS stratified by gender. Corresponding to each test, we report odds ratios and 95% confidence intervals (CIs). First, we fitted unadjusted models. We then fitted multivariable models adjusting for confounding covariates, including offspring’s age in years, education (< high school, high school, college graduate or higher), smoking (2 level variable, smokers/non smokers), physical activity scores, and alcohol use (2 level variable, yes/no). Additional logistic regression was carried out to examine the association of each component of parental MetS (elevated blood pressure, obesity, low HDL-C, high total cholesterol level and impaired fasting glucose) with offspring’s MetS. Multivariable models were used to control for confounders using the above covariates.

## Results

Our study participants included 565 male offspring or sons and 628 female offspring or daughters. Table 1 shows the characteristics of the participants by gender. The mean age was almost similar for male and female offspring members (the mean age was 52.57 ± 8.86 for male and 53.33 ± 9.60 for female). Male offspring were however, more educated and earned more than female offspring. About 14% of female had college education compared to 26% of male participants. Approximately one quarter of the male had a yearly income of $ 45,000 or above. While only 2% female participants’ earning was in that range. Male participants consumed more alcohol than female (76.9% of male and 66.5% of female consumed alcohol, p value < 0.001) and were more physically active than female participant (mean physical activity score 36.19 ± 7.5 for male and 33.81 ± 4.6 for female, p value < 0.001). Male offspring had higher waist circumference, serum triglyceride, fasting blood glucose, resting blood pressure and lower serum HDL-C than female offspring (p value <0.05 for all except resting systolic blood pressure). Over all, about 43% of the male offspring and 35.5% of the female offspring had MetS (p value < 0.01). In terms of individual components, male offspring had higher prevalence of elevated blood pressure (65.3% versus 57.8%, p value < 0.01), elevated triglyceride (54.3% versus 43.2%, p value < 0.001), IFG (32.0% versus 16.2%, p value < 0.001) and low HDL-C (47.4% versus 41.4%, p value < 0.05), than female offspring. Female offspring however, had more abdominal obesity than male members (66.4% versus 49.4%, p value < 0.001). Table 2 shows the distribution of parental clinical characteristics by offspring’s gender. Mothers (mean age 69.47 years for male and 70.49 for female offspring) were slightly older than fathers (mean age 67.2 years for male and 68.36 for female offspring). The prevalence of mother’s obesity and total cholesterol level were higher than father’s prevalence of obesity and cholesterol level for both male and female offspring. On the other hand, prevalence of father’s elevated blood pressure, impaired blood glucose and low HDL-C were higher than mother’s prevalence of elevated blood pressure, impaired blood glucose and low HDL-C for male and female offspring. However, overall, the clustering of the risk factors or the prevalence of maternal MetS was higher than paternal MetS for both, male (prevalence was 25.5% for maternal and 14.6% for paternal MetS) and female (prevalence was 28.9% for maternal and 17.5% for paternal MetS) offspring. About 4.9% of male and about 7.7% of female offspring’s both parents had MetS.

**Table 1:** **Characteristics of the 565 male and 628 female offspring who had information about either parent’s metabolic syndrome status in the Framingham Heart Study (N = 1193)**

	Male (n = 565)	Female (n = 628)	P value^a^
Age	52.57 (8.86)	53.33 (9.60)	0.537
Education status, %			<0.001
High school	34.3	38.4	
College	39.5	47.5	
More than college	26.2	14.1	
Number of years of school completed	14.35 (2.24)	13.91 (2.02)	<0.01
Income Level, %			<0.001
< $20,000,	11.1	63.2	
$20,000- $44,000,	63.4	34.7	
$45,000 or higher	25.5	2.1	
Smoked cigarettes regularly last year, %^b^	17.0	9.0	0.219
Alcohol consumption, %^c^	76.9%	66.5%	<0.001
Physical activity Score^d^	36.19 (7.51)	33.81 (4.61)	<0.001
Waist circumference (cm)	40.58 (4.43)	37.64 (5.78)	<0.001
Serum triglyceride level (mmol/l)	1.54 (0.63)	1.42 (0.41)	<0.05
Serum HDL-C level (mmol/l)	1.19 (0.33)	1.51 (0.40)	<0.001
Resting systolic blood pressure	125.74 (17.41)	124.89 (20.52)	0.468
Resting diastolic blood pressure	75.81 (9.66)	72.58 (9.92)	<0.001
Fasting blood glucose level	5.99 (1.58)	5.47 (1.14)	<0.001
Abdominal obesity%^e^	49.4	66.4	<0.001
High Triglyceride%^f^	54.3	43.2	<0.001
Low HLD-C%^g^	47.4	41.4	<0.05
Elevated blood Pressure%^h^	65.3	57.8	<0.01
Impaired fasting glucose%^i^	32.0	16.2	<0.001
Metabolic syndrome%^j^	43.0	35.5	<0.01

Data presented as mean (SD) or percentage of subjects.

^a^Independent t test or Chi-square test.

^b^Smokers if they had smoked regularly in the previous year.

^c^Alcohol use was dichotomized on the basis of consumption of at least one drink per week.

^d^Calculated based on the average daily number of hours of sleep and sedentary, slight, moderate, and heavy activity of the participant

^e^waist circumference > 102 cm for men and > 88 cm for women

^f^fasting plasma triglyceride concentration ≥ 1.7 mmol/l or on drug treatment

^g^HDL cholesterol levels (<1.03 mmol/l for men and < 1.3 mmol/l in women or on drug treatment.

^h^Blood pressure ≥ 130 mm Hg systolic or ≥ 85 mm Hg diastolic or on drug treatment.

^i^fasting glucose ≥ 6.1 mmol/l or on drug treatment.

^j^Metabolic Syndrome defined as having at least three of the following five components: (1) abdominal obesity or large waist circumference (>102 cm for men and > 88 cm for women); (2) high triglyceride levels (fasting plasma triglyceride concentration ≥ 1.7 mmol/l or on drug treatment); (3) low HDL cholesterol levels (<1.03 mmol/l for men and < 1.3 mmol/l in women or on drug treatment); (4) elevated blood pressure (≥130 mm Hg systolic or ≥ 85 mm Hg diastolic or on drug treatment); or, (5) IFG or impaired fasting glucose (≥6.1 mmol/l or on drug treatment).

**Table 2:** **Parental characteristics of the 565 male and 628 female offspring in the Framingham Heart Study (N = 1193)**

	Male (n = 565)	Female (n = 628)	P value^a^
Mother’s age in years	69.47 (8.01)	70.49 (7.86))	0.07
Father’s age in years	67.2 (7.52)	68.36 (6.40)	0.34
Mother’s obesity %^b^	23.9	25.7	0.29
Father’s obesity %	20.5	22.7	0.28
Mother’s high cholesterol %^c^	63.0	61.5	0.35
Father’s high cholesterol %	33.7	37.2	0.12
Mother’s low HLD-C %^d^	36.8	36.6	0.50
Father’s low HLD-C %	45.5	42.9	0.26
Mother’s elevated blood pressure %^e^	85.3	85.7	0.47
Father’s elevated blood pressure %	87.5	88.5	0.31
Mother’s impaired blood glucose %^f^	6.6	8.9	0.11
Father’s impaired blood glucose %	10.1	8.7	0.31
Mother’s metabolic syndrome %^g^	25.5	28.9	0.26
Father’s metabolic syndrome %	14.6	17.5	0.31
Both parents’ metabolic syndrome %	4.9	7.7	0.21

Data presented as mean (SD) or percentage of subjects.

^a^Independent t test or Chi-square test.

^b^body mass index ≥ 30.

^c^total cholesterol level ≥ 6.20 mmol/l and/ or use of cholesterol-lowering medications.

^d^HDL cholesterol levels (<1.03 mmol/l for men and < 1.3 mmol/l in women or on drug treatment.

^e^Blood pressure ≥ 130 mm Hg systolic or ≥ 85 mm Hg diastolic or on drug treatment.

^f^random blood glucose level ≥ 11.1 mmol/l or if they used insulin or oral hypoglycemic agents.

^g^Metabolic Syndrome defined as having at least three of the following five components: (1) body mass index ≥ 30; (2) total cholesterol level ≥ 6.20 mmol/l and/or use of cholesterol-lowering medications; (3) low HDL cholesterol levels (<1.03 mmol/l for men and < 1.3 mmol/l in women or on drug treatment); (4) elevated blood pressure (≥130 mm Hg systolic or ≥ 85 mm Hg diastolic or on drug treatment); or, (5) random blood glucose level ≥ 11.1 mmol/l or if they used insulin or oral hypoglycemic agents.

Table 3 presents the characteristics of participants by MetS status. Men had higher MetS prevalence than women. Participants who had MetS were older (mean age 55.06 versus 51.63, p value <0.001), were less educated (14.4% with MetS went to more than college versus 23.2% without MetS, p value < 0.01), more likely to be smoker (24.3% with MetS smoked versus 19.4% without MetS, p value < 0.05), and less likely to consume alcohol (64.2% with MetS consumed alcohol versus 76.1% without MetS, p value < 0.001)

**Table 3:** **Characteristics of the participant by metabolic syndrome status in the Framingham Heart Study (N = 1193)**

	Metabolic syndrome (N = 466)	No metabolic syndrome (N = 727)	P value^a^
Age	55.06 (8.62)	51.63 (9.42)	<0.001
Sex %			<0.01
Men	52.1	44.3	
Women	47.9	55.7	
Education status %			<0.01
High school	41.8	33.2	
College	43.8	43.6	
More than college	14.4	23.2	
Number of years of school completed	13.80 (2.06)	14.31 (2.15)	<0.001
Income level %			0.39
< $20,000,	38.5	36.8	
$20,000- $44,000	49.8	48.2	
$45,000 or higher	11.7	15.0	
Smoked cigarettes regularly last year %^b^	24.3	19.4	<0.05
Alcohol consumption %^c^	64.2	76.1	<0.001
Physical activity score^d^	34.67 (6.53)	35.09 (6.04)	0.27

Data are mean (SD) or percentage of subjects.

^a^Independent t test or Chi-square test.

^b^Smokers if they had smoked regularly in the previous year.

^c^Alcohol use was dichotomized on the basis of consumption of at least one drink per week.

^d^Calculated based on the average daily number of hours of sleep and sedentary, slight, moderate, and heavy activity of the participant.

The association between parental and offspring’s MetS are presented in Table 4. After adjusting for age, education, smoking, alcohol consumption and physical activity level of offspring, no significant association was found between father’s and their offspring’s MetS. Mother’s MetS was significantly and positively associated with their female offspring or daughters’ MetS. Daughters with mother’s MetS had 63% (adjusted odds ratio or adj OR: 1.63; 95% confidence Interval, CI:1.02-2.61) increased odds of developing MetS compared to daughters whose mothers didn’t have met syndrome. No significant relationship was noted between mother’s MetS and their male offspring or their son’s MetS. Offspring were however at increased risk if both parents had MetS. Daughters (adj OR: 3.64; 95% CI:1.11-13.07) and sons (adj OR: 4.21; 95% CI: 0.69-25.9) both had about 4 folds of increased odds of having MetS if both parents had MetS compared to offspring whose neither or single parent had MetS.

**Table 4:** **Association between parental and offspring’s metabolic syndrome in Framingham Heart Study (N = 1193)**

Metabolic syndrome		Metabolic syndrome			
		Female offspring or daughters (N = 628)		Male offspring or sons (N = 565)	
		Odds ratio (95% CI)	P value	Odds ratio (95% CI)	P value
Mother	Unadjusted	1.96 (1.31-2.93)	0.001	1.26 (0.81-1.94)	0.30
	Adjusted^a^	1.63 (1.02-2.61)	0.04	1.17 (0.70-1.94)	0.54
Father	Unadjusted	1.24 (0.67-2.32)	0.50	0.97 (0.50-1.89)	0.86
	Adjusted^a^	1.21 (0.59-3.12)	0.19	1.12 (0.53-2.50)	0.72
Both parents	Unadjusted	3.96 (1.23-9.77)	0.01	4.88 (1.20-20.71)	0.02
	Adjusted^a^	3.64 (1.11-13.07)	0.015	4.21 (0.69-25.9)	0.11

Metabolic Syndrome defined as having at least three of the following five components: (1) abdominal obesity or large waist circumference (>102 cm for men and > 88 cm for women); (2) high triglyceride levels (fasting plasma triglyceride concentration ≥ 1.7 mmol/l or on drug treatment); (3) low HDL cholesterol levels (<1.03 mmol/l for men and < 1.3 mmol/l in women or on drug treatment); (4) elevated blood pressure (≥130 mm Hg systolic or ≥ 85 mm Hg diastolic or on drug treatment); or, (5) IFG or impaired fasting glucose (≥6.1 mmol/l or on drug treatment).

^a^The multivariate models are adjusted for age, education, smoking, alcohol consumption, physical activity score.

We estimated the association between each individual risk factor of parents and the presence of MetS among their offspring in Table 5. When analyzed by individual components, the adjusted analysis showed a similar pattern of association between mothers and daughters that we observed previously with the combination or clustering of risk factors. Individual risk factors of mothers were also significantly associated with presence of MetS in their daughters as clustering of the risk factors was. Mother’s impaired blood glucose (adj OR: 2.03; 95% CI: 1.02- 9.31), obesity (adj OR: 1.58; 95% CI: 0.98- 2.56) and low HDL-C (adj OR: 2.01; 95% CI: 1.36-3.32) were independently associated daughter’s MetS. None of these were significant for their son’s MetS. But maternal raised total cholesterol was marginally associated with their son’s MetS (adj OR: 1.60; 95% CI: 0.99-2.58). For fathers, only impaired blood glucose (adj OR: 4.91; 95% CI: 1.77- 11.67) was significantly associated with their daughter’s MetS. None of individual risk factors in fathers was significantly associated with their son’s MetS

**Table 5:** **Association between cardiovascular risk factor in parents and the presence of metabolic syndrome in their offspring in Framingham Heart Study (N = 1193)**

Risk factors		Metabolic syndrome			
		Female offspring N = 628		Male offspring N = 565	
		Odds ratio (95% CI)	P value	Odds ratio (95% CI)	P value
**Mother**					
Elevated BP^b^	Unadjusted	2.04 (1.16-3.58)	0.01	1.77 (1.01-3.11)	0.04
	Adjusted^a^	1.42 (0.71-2.8)	0.32	1.28 (0.67-2.45)	0.46
Impaired BG^c^	Unadjusted	3.15 (1.61-6.17)	0.001	0.801 (0.37-1.7)	0.57
	Adjusted^a^	2.03 (1.02- 9.31)	0.04	0.40 (0.18-1.22)	0.12
Obesity^d^	Unadjusted	1.81 (1.19-2.74)	0.005	1.596 (1.02-2.5)	0.04
	Adjusted^a^	1.58 (0.98-2.56)	0.06	1.41 (0.83-2.39)	0.20
Raised total-C^e^	Unadjusted	1.15 (0.79-1.67)	0.475	1.49 (1.00-2.22)	0.05
	Adjusted^a^	1.12 (0.72-1.80)	0.38	1.60 (0.99-2.58)	0.05
Low HDL-C^f^	Unadjusted	2.05 (1.40-3.00)	0.000	1.39 (0.89-1.95)	0.15
	Adjusted^a^	2.01 (1.36-3.32)	0.001	1.47 (0.93-2.36)	0.09
**Father**					
Elevated BP	Unadjusted	1.21 (0.54-2.74)	0.64	1.37 (0.64-2.90)	0.41
	Adjusted^a^	0.88 (0.32-1.84)	0.48	1.62 (0.65-4.00)	0.24
Impaired BG	Unadjusted	3.35 (1.60-7.02)	0.001	1.36 (0.60-2.66)	0.53
	Adjusted^a^	4.91 (1.77-11.67)	0.001	1.38 (0.56-3.36)	0.48
Obesity	Unadjusted	1.29 (0.73-2.28)	0.39	1.08 (0.61-1.92)	0.79
	Adjusted^a^	1.81 (0.87-3.6)	0.12	1.31 ( 0.67-2.67)	0.4
Raised total-C	Unadjusted	1.33 (0.81-2..21)	0.26	0.93 (0.56-1.54)	0.78
	Adjusted^a^	1.42 (0.79-2.68)	0.22	0.83 (0.45-1.52)	0.54
Low HDL-C	Unadjusted	1.53 (0.84-2.77)	0.16	1.43 (0.81-2.42)	0.22
	Adjusted^a^	1.48 (0.73-2.98)	0.27	1.45 (0.77-2.74)	0.24

BP: Blood pressure, BG: Blood glucose, Total-C: Total Cholesterol, HDL-C: HDL cholesterol.

^a^The multivariate models are adjusted for age, education, smoking, alcohol consumption, physical activity score.

^b^Blood pressure ≥ 130 mm Hg systolic or ≥ 85 mm Hg diastolic or on drug treatment.

^c^random blood glucose level ≥ 11.1 mmol/l or if they used insulin or oral hypoglycemic agents.

^d^body mass index ≥ 30.

^e^total cholesterol level ≥ 6.20 mmol/l and/or use of cholesterol-lowering medications.

^f^HDL cholesterol levels (<1.03 mmol/l for men and < 1.3 mmol/l in women or on drug treatment.

## Discussion

Using the parents and offspring data from FHS, we found differential association of MetS and its components between parents and offspring. In our study, maternal MetS was significantly associated with daughter’s MetS, but not with son’s MetS. Individual maternal risk factors like impaired blood glucose, obesity, and low HDL-C were also independently associated with daughter’s MetS. Maternal low HDL-C and raised total cholesterol showed marginal association with son’s MetS. The relationship between father-offspring in regard to the presence of MetS was however, relatively weaker and didn’t reach conventional level of significance. Only father’s impaired blood glucose had significant association with their daughter’s MetS.

It is widely accepted that cardiovascular disease and risk factors are heritable through generations from parents to offspring and intergenerational associations of individual cardiovascular risk factors have been reported in a number of studies [18–29]. However, to our knowledge, very few studies have measured the association of MetS or clustering of risk factors between parents and offspring. One study conducted in Korean population found a strong parent–offspring association for MetS between adolescents and their parents [6]. However, they didn’t report on differential transmission from mothers or fathers. The only work that we found reporting differential association is from Fels Longitudinal Study data [7]. Unlike the current study, the FeLs study showed a significant association between sons, and both mothers and fathers, but a weaker association between daughters and mothers. One of the reasons behind this difference in the results could be the inclusion of young aged offspring in their analysis. As the onset of cardiovascular disease and cardiovascular risk factors is usually later in female than in male, probably inclusion of young aged offspring (18–32 years) masked the relationship between daughter and mothers in the Fels Study when MetS or clustering of risk factors was considered due to low prevalence of MetS among young daughters [30]. But in the same study, maternal presence of MetS was significantly and positively related with individual risk factors like blood pressure and triglycerides in female offspring indicating that with progress of age, mother-daughter association in regard to clustering could become significant. Also this study didn’t adjust for important lifestyle or socio-demographic characteristics such as, alcohol and smoking behavior, level of physical activity, and education. The stronger parent- son association that they found might have become weaker if those factors were accounted for. Among the individual components of MetS, to date, researchers have mostly focused on measuring intergenerational transmission of obesity and insulin resistance or diabetes. A number of longitudinal and observational studies have shown stronger maternal–offspring transmission of type 2 diabetes [18–20]. But the evidence on differential transmission of obesity or BMI is mixed. Some studies have reported a stronger maternal than paternal parent offspring association of BMI and obesity [21–24], whereas others have showed similar associations [25–28].

The parental transmission of different risk factors or clustering of risk factors can be attributed to both genetic and environmental factors. We adjusted for important lifestyle and socio-demographic characteristics such as, smoking and alcohol consumption, level of physical activity, and education to estimate the accurate parent-offspring association of MetS and its components. These adjustments attenuated the risk estimates. But a significant association that remained even after the adjustment, mainly with maternal MetS and with maternal risk factors, suggests that this specific maternal effect could be partly attributed to a direct effect of maternal uterine environment on the development of fetus. During the past few decades, particular attention has been given to maternal health and exposure during pregnancy influencing the future health of the offspring. It is now believed by many scientists that intrauterine conditions can alter the structure and function of organs and physiological systems in the fetus, and can be a cause for subsequent adult disease like adulthood hypertension, insulin resistance and an abnormal lipid profile [31–36]. This alteration of physiological systems can originate through adaptive processes, which the fetus makes when the environment in intrauterine life is unfavorable. This unfavorable condition can be caused by different environmental stimuli like, maternal under nutrition, maternal stress, maternal exposure to toxins, maternal genes affecting placental function, etc. [37]. Differential association of risk factors may also possibly be influenced by differential gene expression between men and women. Scientists have identified several chromosomal regions that are sex-specifically involved in influencing different traits of metabolic syndrome [38]. An increasing body of evidence also supports the role of environmentally-induced epigenetic changes in disease susceptibility. Experimental studies in mice suggest a role for maternal diet in inducing epigenetic changes in the offspring [39, 40]. This would make the mother’s influence on offspring greater than the father’s influence, as our finding indicates.

Besides being part of this biological interaction, maternal dietary habit itself can also have an independent role to play. A research done on nationally representative US sample showed that resemblance in diet between mother–daughter was significantly stronger compared to their father–child counterparts [41]. Thus, if the dietary habit of mothers with MetS is unhealthy, the chance of inheriting that poor dietary habit of mother is increased among daughters compared to their sons. It is also possible that maternal feeding strategies act as a contributor to these effects. In one study obese mothers reported significantly less control over their children's food intake [42]. There can be sex specific adoption of other behaviors of parents like, physical activity, smoking etc. and this can also cause differential associations of risk factors that we found [43, 44].

Our results should be interpreted within the context of few limitations and strength. We acknowledge the considerable disagreement over the definition and diagnostic criteria related to MetS. Of the various available definitions, we used the ATPIII criteria as this is the most widely used definition in the US [1, 45]. It can be argued that some other available definition of MetS could be equally valid and produce somewhat different result. But it is largely believed that metabolic syndrome, however defined, remains pretty similar in prevalence and in risk prediction for Caucasian population [46, 47]. Thus, we are confident that our estimations are pretty accurate. It should be mentioned that we used some proxy measures for parents to define MetS. BMI was used instead of WC, and as parent cohort had non fasting blood, we used total cholesterol level instead of triglyceride and fasting sugar level of ≥11.1 mmol/l for defining insulin resistance. Though all of them are valid substitutes [2, 48], by using these surrogate measures, we might have introduced a non-differential exposure misclassification bias in our study. This possibly has increased the similarity between the exposed and non-exposed groups, and has underestimated the associations in our study [49]. However, there is a very slim possibility that the underestimation was substantial, as we found the similar pattern of parent–offspring association when we estimated the risk of offspring’s MetS by individual parental risk factors. The Framingham cohorts are almost exclusively white and one of the limitations of study includes its inability to generalize the findings to other ethnic groups. The generalizability of the study might be little limited, but this large community based study allowed us avoid systematic errors introduced by self-reported health status of parents. Most of the studies reporting parent-offspring cardiovascular risk factors association depend on self-reported parental health information and are prone to this error. Because we used objectively measured parental anthropometric and laboratory data, the current analysis remained free from this bias. Another strength of the study was that we included adult offspring for our analysis when they didn’t share household with their parents. This improved the precision of our analysis as the calculated estimates were independent of shared familial environmental factors.

Our demonstration of associations between maternal and offspring’s MetS suggests that interventions to reduce the prevalence of MetS aimed at family level and earlier in life are likely to be beneficial. Interventions should be targeting on weakening the link between maternal MetS and offspring’s MetS and its components. This can be achieved by warning parents with MetS, mothers in particular, about their offspring’s increased risk of getting MetS and by encouraging them to promote healthy lifestyle at family level for themselves and for their children. Our findings support the proposition that maternal effect is transmitted through intrauterine mechanisms. From public health point of view, this suggests that adulthood diseases could be prevented by improving maternal health and fetal development. This inspires future research to explore the relationships between different conditions of prenatal, natal and post natal life. Identifying the common mechanisms and pathways involved in these relationships will help to determine the issues to optimize maternal health, birth outcome, and lifelong health of the offspring. Our findings also indicate that family history about multiple risk factors in parent, especially in mothers can add value in offering prognosis and risk assessment. If there is a positive family history of MetS, individual risk factors such as hypertension, obesity, or hypercholesterolaemia should be treated more aggressively so that clustering of the risk factors are prevented or delayed.

## Conclusion

We found differential association of MetS and its components between parents and offspring. Mother’s MetS was strongly related with daughter’s MetS, but the association was inconsistent with son’s MetS. However, no association was found between father’s MetS and offspring’s Mets. We recommend further studies to examine the effects of early life environments and epigenetics on adults MetS. We also think policies and health educational programs should be directed towards families, in particular towards women for future CVD risk reduction.
